# Culturable Diversity of Thraustochytrids from Coastal Waters of Qingdao and Their Fatty Acids

**DOI:** 10.3390/md20040229

**Published:** 2022-03-28

**Authors:** Mohan Bai, Biswarup Sen, Shuai Wen, Huike Ye, Yaodong He, Xiaobo Zhang, Guangyi Wang

**Affiliations:** 1 College of Life Sciences, Zijingang Campus, Zhejiang University, Hangzhou 310058, China; bmh@zju.edu.cn; 2 Center for Marine Environmental Ecology, School of Environmental Science and Engineering, Tianjin University, Tianjin 300072, China; bsen@tju.edu.cn (B.S.); wenshuai2018@tju.edu.cn (S.W.); yehuike@tju.edu.cn (H.Y.); yaodong.he@tju.edu.cn (Y.H.); 3 Key Laboratory of Systems Bioengineering (Ministry of Education), School of Chemical Engineering and Technology, Tianjin University, Tianjin 300072, China; 4 Center for Biosafety Research and Strategy, Tianjin University, Tianjin 300072, China

**Keywords:** thraustochytrids, diversity, biomass, fatty acids, seasons, habitats

## Abstract

Thraustochytrids have gained significant attention in recent years because of their considerable ecological and biotechnological importance. Yet, the influence of seasons and habitats on their culturable diversity and lipid profile remains poorly described. In this study, a total of 58 thraustochytrid strains were isolated from the coastal waters of Qingdao, China. These strains were phylogenetically close to five thraustochytrid genera, namely *Botryochytrium*, *Oblongichytrium*, *Schizochytrium*, *Thraustochytrium*, and *Sicyoidochytrium*. Most of the isolated strains were classified into the genera *Thraustochytrium* and *Oblongichytrium*. Further diversity analysis revealed that samples collected from nutrient-rich habitats and during summer/fall yielded significantly higher culturable diversity of thraustochytrids than those from low-nutrient habitats and winter/spring. Moreover, sampling habitats and seasons significantly impacted the fatty acid profiles of the strains. Particularly, the *Oblongichytrium* sp. OC931 strain produced a significant amount (153.99 mg/L) of eicosapentaenoic acid (EPA), accounting for 9.12% of the total fatty acids, which was significantly higher than that of the previously reported *Aurantiochytrium* strains. Overall, the results of this study fill the gap in our current understanding of the culturable diversity of thraustochytrids in the coastal waters and the impact of the sampling habitats and seasons on their capacity for lipid accumulation.

## 1. Introduction

Microorganisms are recognized as an alternative potential source of omega-3 polyunsaturated fatty acids (PUFAs) [1,2] and saturated fatty acids (SFAs) [3,4]. Many oleaginous microorganisms including microalgae, bacteria, yeast, and fungi have been used as promising feedstocks for lipid production due to their excellent capacities for substrate consumption and lipid production, and their fast growth rates [5,6,7]. Among various types of oleaginous microorganisms, thraustochytrids, a group of unicellular heterotrophic marine protists with ubiquitous presence in coastal environments [8,9], are the most promising because of their fast growth rate and capacity to accumulate a high amount of docosahexaenoic acid (DHA, C22:6n − 3) on diverse substrates (e.g., glucose, glycerol, and food-waste hydrolysate) [10,11]. However, the availability of high-yielding strains remains one of the major limiting factors for large-scale lipid production from thraustochytrids.

The content and composition of lipid vary considerably among different thraustochytrid genera/species, suggesting their different biotechnological potentials. For example, among the three genera (*Schizochytrium*, *Aurantiochytrium*, and *Thraustochytrium*) of thraustochytrids isolated from the coastal waters of Southern China, the DHA yields of *Thraustochytrium* strains were less than 25% of the other two genera [12]. The myristic acid (C14:0) content of *Thraustochytrium* sp. ATCC 26185 was nearly 10-fold higher than that of *Thraustochytrium* sp. ATCC 20891 [13]. Furthermore, different seasons and habitats have been reported to affect the production potential and profiles of fatty acids produced by thraustochytrid strains belonging to the same genus/species. For instance, the *Aurantiochytrium* strains isolated from winter samples were reported to produce about 15% higher PUFAs than those from summer samples [14]. *Aurantiochytrium* strains isolated from Antarctic seawaters showed significantly (usually more than 10-fold) higher eicosapentaenoic acid (EPA) content than those isolated from tropical mangroves [15]. Nevertheless, most of the earlier studies were focused on the isolation of thraustochytrids from mangrove habitats and many of the isolates in those studies were members of the genera Aurantiochytrium and Schizochytrium [16,17]. Only a few studies reported the seasonal succession of culturable thraustochytrids in estuarine and coastal waters [18]. Besides, the lipid profiles of the previously reported estuarine or coastal thraustochytrid strains are poorly described.

In this study, we investigated the culturable diversity of thraustochytrids in the seawaters from three temperate coastal habitats (estuarine, nutrient-rich, and nutrient-poor) of Qingdao, China, across four seasons (summer, autumn, winter, and spring). In addition, the effects of habitats and seasons on the production of biomass and fatty acids were investigated. The results of this study provide an interesting insight into the future bioprospecting of thraustochytrid strains for the production of SFAs and PUFAs.

## 2. Results and Discussion

### 2.1. Phylogenetic Diversity of the Isolates

**Figure 1:**
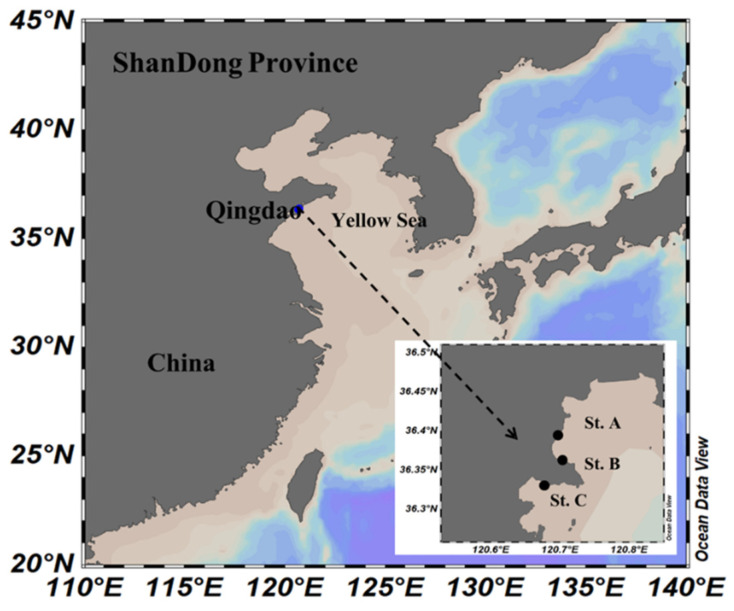
Map of sampling stations in the coastal region of Qingdao, China.

Using the pine-pollen baiting method, more than 400 strains were isolated from the coastal water of Northern China across different seasons and habitats (Figure 1). The colonies of these isolates were mostly whitish in appearance. Under the microscope, their cells generally exhibited globose or sub-globose shape (Figure S1), which was consistent with the typical shape of thraustochytrid cells described in previous studies [12,16]. Partial 18S rRNA gene-sequencing analyses further confirmed these isolates to be thraustochytrids. After deduplicating the isolates of each sample, a total of 58 thraustochytrid strains were finally obtained (Table 1).

**Figure 2:**
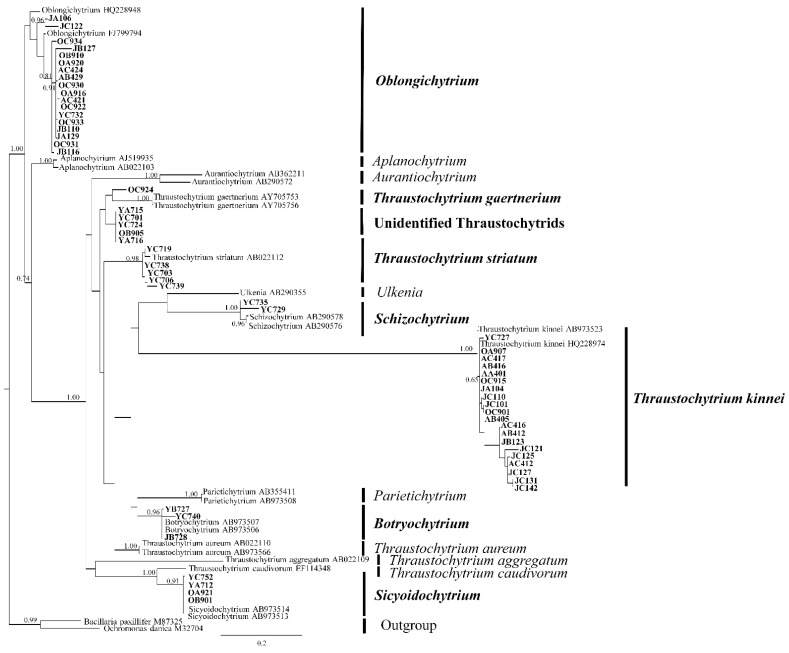
A maximum-likelihood phylogenetic tree of the newly-isolated thraustochytrid strains. The first and second letters of the strains’ label indicate the sampling season (A: spring; Y: summer; O: autumn; J: winter) and location (A: St. A; B: St. B; C: St. C), respectively.

The 58 strains belonged to five different genera, namely *Thraustochytrium*, *Oblongichytrium*, *Sicyoidochytrium*, *Schizochytrium*, and *Botryochytrium* (Figure 2). Five strains (Thraustochytriaceae sp. YC701, Thraustochytriaceae sp. YA715, Thraustochytriaceae sp. YC724, Thraustochytriaceae sp. OB905, and Thraustochytriaceae sp. YA716) formed a phylogenetic group with no previously identified strains, suggesting that they possibly belong to a new genus. Among the five classified genera, *Thraustochytrium* and *Oblongichytrium* were the most dominant genera, accounting for about 44% and 31% of the total isolates, respectively. Previously, members of the genera *Thraustochytrium* and *Oblongichytrium* have been reported to account for 15–20% of the total thraustochytrid OTUs (operational taxonomic units) in the seawaters of the Bohai Sea and Piver’s Island using culture-independent methods [9,19]. Our findings are consistent with these earlier reports, suggesting *Thraustochytrium* and *Oblongichytrium* as the two dominant genera in the coastal waters.

In this study, none of the isolates were phylogenetically related to the genera *Aplanochytrium* and *Aurantiochytrium*. However, *Aplanochytrium* accounted for 24% and 33% of the total thraustochytrid OTUs in the coastal waters of the Bohai Sea and the South China Sea, respectively [8,9]. Members of *Aplanochytrium* have been described as symbiotic or parasitic with zooplankton and seagrasses and were mostly isolated using the direct plating method [20,21,22,23]. Similarly, previous studies reported that more than 60% of the culturable isolates from mangrove habitats belong to the members of *Aurantiochytrium* [12,14,16,24]. These data suggest that the pine pollen baiting method may be unsuitable for isolating members of *Aplanochytrium* and that mangroves may be a favorable habitat for the members of *Aurantiochytrium*.

### 2.2. Season- and Habitat-Specific Culturable Diversity

The culturable diversity of thraustochytrids comprised five known genera (*Thraustochytrium*, *Botryochytrium*, *Oblongichytrium*, *Sicyoidochytrium*, and *Schizochytrium*) and unclassified thraustochytrids in the summer, three known genera (*Oblongichytrium*, *Sicyoidochytrium*, and *Thraustochytrium*) and unclassified thraustochytrids in autumn, and two known genera (*Oblongichytrium* and *Thraustochytrium*) in winter and spring (Table 1). This result indicated that low temperature was likely one of the main factors that limited the culturable genera of thraustochytrids. Previous studies have also reported temperature as the main factor that regulated the culturable genera/species of fungi and yeast, and at a low temperature, only a few genera/species dominated [25,26,27]. Furthermore, temperature is one of the most important factors influencing the growth and metabolic potential of thraustochytrids [28]. However, the effect of cultivation temperature on the isolation of thraustochytrids under laboratory culture conditions remains unclear. In our study, a total of 22 strains could be isolated under 28 °C as the cultivation temperature. Some previous studies had set the culture temperature at 5–20 °C to obtain more isolates from cold environments [15,29]. However, a few studies that used 20 °C and 25 °C as the cultivation temperatures could isolate a total of only 22 [15] and 35 [29] strains from samples collected in cold seasons, respectively. Therefore, the high culture temperature (28 °C) set in our study may not likely underestimate the culturable genera of thraustochytrids in the winter. Furthermore, only nutrient-rich habitats (St. C) had lower numbers of strains in winter than in summer, indicating that the isolation of high lipid accumulating thraustochytrids strains is not limited by the season.

**Figure 3:**
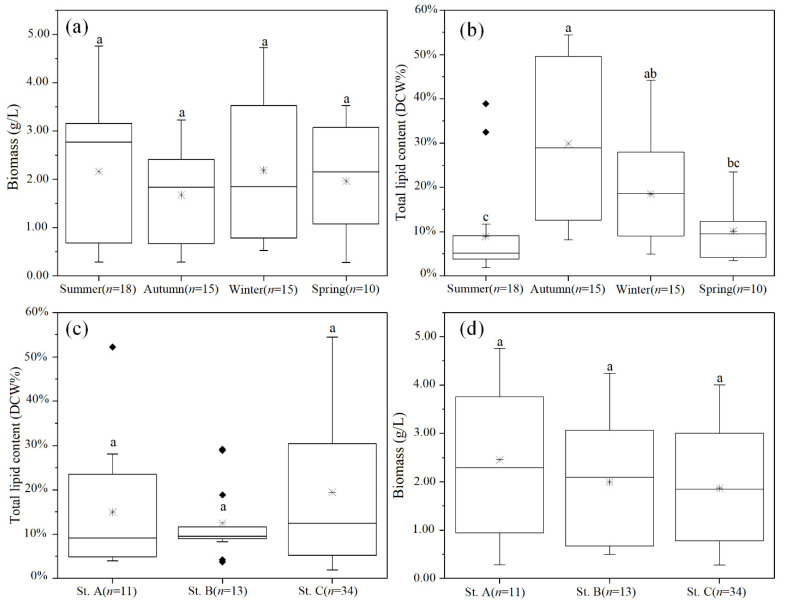
Biomass and lipid content of thraustochytrid strains isolated from coastal waters across different (**a**,**b**) seasons and (**c**,**d**) habitats. The number of strains in each group is represented by “*n*”. The “*” inside each box represents the median and the “♦” represent the outliers. Statistical significance was analyzed by Dunn’s test after the Kruskal–Wallis test. The significant differences are indicated by different letters.

Among the three sampling stations, the highest number of strains (i.e., 34 strains) was isolated from the nutrient-rich waters of St. C (Table 1). The numbers of strains isolated from the nutrient-poor waters of St. B and estuarine waters of St. A were 13 and 11, respectively (Table 1). The isolation of many strains from St. C possibly indicates the increased chances of adsorption of thraustochytrid cells on pine pollen in nutrient-rich environments. Furthermore, the isolates from St. C belonged to five known genera (Figure 3b), suggesting more culturable diversity of thraustochytrids in nutrient-rich coastal waters than that in nutrient-poor coastal waters. Our findings are consistent with a previous study that reported higher diversity of thraustochytrids in the nutrient-rich waters than that in the nutrient-poor waters along the coastal region of Japan [18]. Some earlier studies [8,18,30], which reported a positive correlation between the abundance of thraustochytrids and the nutrient levels in the coastal waters, further support our results.

Taken together, the findings of this study suggest high culturable diversity of thraustochytrids in the coastal waters under summer temperature and nutrient-rich conditions. Our study provides information that would guide future efforts on the isolation of thraustochytrids from unexplored regions.

### 2.3. Variations of Biomass and Lipid Contents of Cultured Strains

The cell biomass of the 58 thraustochytrid strains varied between 0.28 g/L and 4.76 g/L (Figure 3a,c), which was comparable to the reports of isolates from Indian mangroves (1.10–3.64 g/L), Malaysian mangroves (1.07–5.82 g/L), and Australian estuarine waters (0.67–3.07 g/L) [24,31]. However, the biomass values of these strains were much lower than those isolated from some other Indian mangroves (Navi Mumbai: 14.12–22.98 g/L; Kerala: 7.3–10.6 g/L) and Chon Buri of Thailand (6.88–22.49 g/L) [14,32,33]. Although the biomass values varied widely among the isolates, any significant (*p* > 0.05, one-way ANOVA) association between biomass and habitats or seasons was not observed in our study. In addition, there was no significant (*p* > 0.05, one-way ANOVA) association between cell biomass and genera (Table S3).

The lipid yield of the 58 strains ranged between 1.9% DCW and 54.5% DCW with an average yield of 17% DCW (Figure 3b,d). These yields were significantly lower than those of the previous thraustochytrid strains, which showed an average yield of 30–40% DCW and occasionally ~70% DCW (Figure 3b,d) [14,32,33]. The observed differences in the yield could be due to the different genera of the strains that were isolated in our study. Most of the strains in this study belonged to *Oblongichytrium* and *Thraustochytrium*, while the strains of previous reports mainly belonged to *Aurantiochytrium*. Notably, the members of the latter genus are well-known for their higher biomass and lipid content [14,32,33,34]. Furthermore, the yields varied significantly (*p* < 0.05, one-way ANOVA) among different seasons (Figure 3b), but not (*p* > 0.05, one-way ANOVA) among different habitats (Figure 3d). The strains isolated from the autumn samples showed significantly (*p* < 0.05, one-way ANOVA) higher lipid yield (29.8% average yield) than the strains isolated from the summer (8.89% average yield) and spring (10.11% average yield) samples (Figure 3b). The higher lipid-yielding strains belonged to the *Oblongichytrium* (36.84% average yield) and *Thraustochytrium* (30.86% average yield) strains, which were isolated from the autumn samples (Table S2). However, the strains that were isolated from the spring and summer samples and belonged to those same genera showed significantly (*p* < 0.05, one-way ANOVA) lower lipid yields (Table S2). These results suggest that strains that are members of the same genera but isolated from different seasons can accumulate significantly different amounts of lipids.

A previous study found that thraustochytrids with higher lipid contents were more likely to survive in low-temperature conditions [35]. Thus, some thraustochytrid strains with lower fatty acid contents may be eliminated during the seasonal temperature changes. Even for the *Aurantiochytrium* strains, which are well-known for their high lipid contents, the strains isolated in winter showed 5–15% higher lipid yield than those isolated in summer [14]. Our results, which revealed seasonal variations in the lipid yields of the *Oblongichytrium* and *Thraustochytrium kinnei* strains, are consistent with the previous studies and suggest that choice of season may be crucial for isolating high-lipid-yielding thraustochytrids.

### 2.4. Fatty Acid Profiles and Their Variations

The fatty acid profiles of the 58 strains revealed that palmitic acid (PA, C16:0, 7.90–37.12%) was the most abundant fatty acid among the saturated fatty acids (SFAs) (Table 2). The proportions of the other SFAs, such as stearic acid (C18:0, 0.43–22.30%), pentadecanoic acid (C15:0, 0.72–12.05%), and arachidic acid (C20:0, 0–13.81%), were relatively lower, while those of dodecanoic acid (C12:0), myristic acid (C14:0), and heptadecanoic acid (C17:0) were ≤2%. Among the detected PUFAs, docosahexaenoic acid (DHA, C22:6n − 3) was the major fatty acid, accounting for 5.37–46.86% of TFAs. Docosapentaenoic acid (DPA, C22:5n − 3) and eicosapentaenoic acid (EPA, C20:5n − 3) were detected in all the strains within a range of 0.43–19.07% and 0.25–15.11%, respectively. In addition, arachidonic acid (C20:4n − 6) was also detected, but its proportion was ≤2%. As C16:0, C22:6, C22:5, and C20:5 are important fatty acids for the biofuel and pharmaceutical industries [36,37,38], the strains isolated in our study could be potential cell factories for industrial applications.

**Figure 4:**
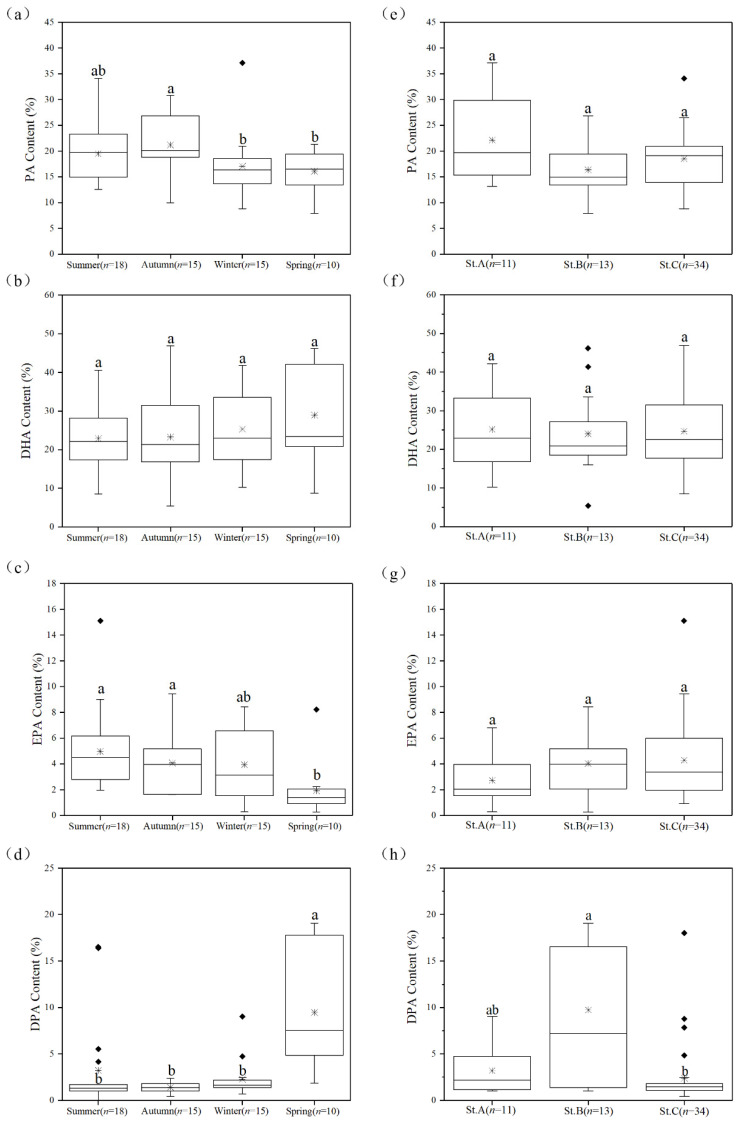
Variations of the proportions of PA, DHA, EPA, and DPA in the new strains isolated from coastal waters across different (**a**–**d**) seasons and (**e**–**h**) habitats. The number of strains in each group is represented by “*n*”. The “*” inside each box represents the median and the “♦” represent the outliers. Statistical significance was analyzed by Dunn’s test after the Kruskal–Wallis test. The significant differences are indicated by different letters. The content of each fatty acid is provided as % of total fatty acids.

Further analysis revealed that strains isolated from the autumn samples showed a significantly (*p* < 0.05, one-way ANOVA) higher average proportion of PA (21.20%) than those of the strains isolated from the winter (17.05%) and spring (16.06%) samples (Figure 4a). Although not statistically significant, the average proportions of DHA in strains isolated from the winter (25.31%) and spring (28.87%) samples were higher than those in strains isolated from the summer (22.87%) and autumn (23.26%) samples (Figure 4b). Likewise, the average proportion of EPA in strains isolated from the spring samples (1.92%) was significantly lower than those of strains isolated from the summer (4.94%), autumn (4.06%), and winter (1.92%) samples (Figure 4c). Conversely, the average proportion of DPA in strains isolated from the spring samples (9.46%) was significantly (*p* < 0.05, one-way ANOVA) higher than those in strains isolated from the summer (3.25%), autumn (1.41% TFAs), and winter (2.28%) samples (Figure 4d). Interestingly, among the major fatty acids (Figure 4e–g), DPA was the only fatty acid whose proportion was affected by both seasons and habitats (Figure 4h). The average proportion of DPA (9.74%) in strains isolated from the nutrient-poor waters (St. B) was three-fold and four-fold higher than those in strains isolated from estuarine (St. A) and nutrient-rich (St. C) waters, respectively.

Previous studies have found higher DHA contents in thraustochytrids isolated from the cold seasons/regions [14,24]. Our results support such findings and further show that DPA, EPA, and PA can also be affected by the sampling seasons. Moreover, the characteristic seasonal variations of the average proportions of these fatty acids observed in our study suggest that future strategies for the isolation of thraustochytrids can be designed depending on the target fatty acid (Figure 4). Furthermore, previous studies have reported extremely low levels of EPA (usually less than 100 mg/L and 2% proportion) in *Aurantiochytrium* strains isolated from mangroves [14,16,33]. Notably, in this study, except for the strains isolated from the spring samples, the proportion of EPA in all other strains was greater than 2%. Particularly, the EPA content of *Oblongichytrium* sp. OC931 was up to 153.99 mg/L accounting for 9.12% of TFAs. These findings indicate that coastal waters may be a better habitat for isolating EPA-rich thraustochytrids compared with the mangrove habitat. More importantly, the EPA production in this study was without any process optimization and, therefore, it is likely that under optimized conditions, the isolate OC931 may produce greater amounts of EPA.

## 3. Materials and Methods

### 3.1. Sample Collection and Isolation of Thraustochytrids

Seawater samples were collected from three different coastal stations (St. A, St. B, and St. C) of Qingdao, China in August 2018 (summer), October 2018 (autumn), January 2019 (winter), and April 2019 (spring) (Figure 1). At each station for each season, 2–3 samples were collected and pooled for isolation of thraustochytrids. St. A was situated close to the Wenquan River estuary and characterized by the lowest salinity level among the three stations (Table S1). St. B was located far away from human activity and contained the lowest level of total nitrogen (69.17 ± 19.05 μg/L) among the three stations, while St. C was in an aquaculture area with the highest level of total nitrogen (114.35 ± 28.69 μg/L) (Table S1).

Surface water at ~2 m depth was collected into sterile plastic tubes and transported to the laboratory for further analysis. The isolation of thraustochytrids was carried out using the pine-pollen baiting method [16]. As thraustochytrids tend to become attracted to and gathered on pine-pollen [39], this method seems to increase the probability of isolating efficient thraustochytrid strains. In brief, a small amount of sterilized pine pollen was added to 5 mL seawater in Petri dishes. The resulting Petri dishes were incubated at 28 °C and the enrichments were monitored daily under a microscope for 3–7 days. The pine pollens attached with potential thraustochytrids were picked and sub-cultured on modified Mar Chiquita (MC) medium (containing 2 g/L glucose, 1 g/L peptone, 1 g/L yeast extract, 1 g/L sodium glutamate, 1 g/L corn steep liquor, 20 g/L agar, and 33 g/L artificial sea salt) amended with antibiotics (0.075% streptomycin, 0.005% nystatin, and 0.05% ampicillin) [40]. The MC plates containing the enriched pollens were incubated at 28 °C until their colonies grew. The resulting colonies were transferred to fresh MC medium containing antibiotics for purification. All the purified isolates were maintained at 4 °C on modified Vishniac’s (MV) medium containing 2 g/L glucose, 1 g/L peptone, 1 g/L yeast extract, 20 g/L agar, and 33 g/L artificial sea salt [20].

### 3.2. Sequencing and Phylogenetic Analysis

The isolates were identified based on their 18S rRNA gene sequences. The isolated strains were cultured in an M4 medium at 28 °C and 150 rpm for 4 days. The total genomic DNA was then extracted using a DNA-extraction kit (Generay, Shanghai, China) following the manufacturer’s protocol. The partial 18S rRNA gene fragments of these strains were amplified by polymerase chain reaction (PCR) using primers 18S001 (AACCTGGTTGATCCTGCCAGTA) and 18S13 (CCTTGTTACGACTTCACCTTCCTCT) in an S1000™ thermal cycler (Bio-Rad, Richmond, CA, USA) [41]. Approximately 5 ng of genomic DNA was used as a template in a 25 μL reaction volume containing 12.5 μL of Taq PCR mix (Takara Bio, Otsu, Japan) and 10 μM of each primer. The PCR program included 95 °C for 5 min, 35 cycles of 60 s at 94 °C, 60 s at 52 °C, and 60 s at 72 °C, with a final extension of 72 °C for 10 min [8]. The resulting PCR products were checked by agarose gel electrophoresis and purified using a gel DNA-extraction kit (TIANGEN, Beijing, China). The purified PCR products were then cloned into TOPO vectors (Aidlab, Beijing, China) and transformed into competent *Escherichia coli* DH5α cells by following the protocol of the TA cloning system (Aidlab, Beijing, China). Clones containing positive insert were sequenced at BGI (Beijing, China) using M13 universal primers. All the sequences generated in this study have been submitted to GenBank with accession numbers of OM418567–OM418624.

Using the MAFFT program (https://www.ebi.ac.uk/Tools/msa/mafft/, accessed on 5 January 2022), the resulting 18S rRNA sequences were compared against the reference sequences available in the NCBI database and were subsequently used for the construction of a maximum-likelihood (ML) phylogenetic tree with RAxML [42]. *Bacillaria paxillifer* (GenBank: M87325) and *Ochromonas danica* (GenBank: M32704) were chosen as outgroups for the phylogenetic tree.

### 3.3. Quantification of Biomass and Fatty Acids

The cell biomass of each strain was determined by the gravimetric method. In brief, an aliquot of culture broth was centrifuged at 10,000× *g* rpm for 20 min at 4 °C. The resulting biomass was washed twice with sterile distilled water. Then, the wet biomass was dried using a freeze-dryer (Christ, Hamburg, Germany) for 48 h. The cell pellets were stored at −80 °C for further fatty acids analysis. The biomass was expressed as dry cell weight (DCW) in g/L.

Analysis of fatty acid composition was carried out using the direct transesterification method [43]. Briefly, 50 mg freeze-dried cells were transferred to 2 mL of 4% sulfuric acid in methanol. One hundred microliters of nonadecanoic acid (C19:0) (1.0 mg/mL) solution was added into the mixture as an internal standard. The resulting mixture was vortexed for 30 s, followed by incubating for 1 h at 80 °C. After the mixture was cooled down to room temperature, 1 mL each of distilled water and n-hexane was added to the mixture, and the resulting mixture was centrifuged at 4000× *g* rpm for 5 min for phase separation. The upper layer of hexane containing fatty acid methyl esters (FAMEs) was collected and analyzed using an Agilent 7890B gas chromatography device (Agilent, Foster City, CA, USA) equipped with a DB-WAX column (60 m × 320 µm × 0.15 µm) and a flame ionization detector (FID). Samples were injected in the split injection mode with a split ratio of 50:1. The injection port temperature was set to 250 °C. Nitrogen was used as the carrier gas with a flow of 1 mL/min. The column temperature was originally set to 50 °C for 1 min, followed by programming at 25 °C/min to 175 °C, then was increased to 220 °C at 3 °C/min and held for 5 min, and finally reached 230 °C at 2 °C/min and was held for 11 min [16]. The fatty acids were identified based on the retention times of the standard mix (Sigma-Aldrich, SaintLouis, MO, USA). The internal standard, C19:0, was added to each sample to quantify the fatty acid components of the sample and improve the precision of the quantitation. The lipid yield was calculated based on the dry cell weight.

### 3.4. Statistical Analysis

One-way ANOVA (Kruskal–Wallis test) and post hoc (Dunn’s test) analysis were performed to test the significant differences of biomass and lipid contents among the seasons, habitats, or genera. The R package “PMCMRplus” was used for performing the Kruskal–Wallis and Dunn’s tests. All statistical analyses were carried out in R software (version 3.4.2, https://www.r-project.org/, accessed on 14 January 2022).

## 4. Conclusions

This study reports the isolation of 58 thraustochytrid strains from the coastal waters of Northern China, their phylogenetic affiliation, and their association with the environmental conditions. Although the strains were phylogenetically related to five different thraustochytrid genera, most of them belonged to the genera *Thraustochytrium* and *Oblongichytrium*. Interestingly, the fatty acid profiles of these strains were significantly affected by the season and habitat of the isolation source. Particularly, some strains showed good potential for EPA production. Overall, this culture-based study expands our current understanding of the culturable diversity of thraustochytrids and provides useful information for designing future isolation strategies and source selection.

## Figures and Tables

**Table 1:** Numbers of thraustochytrid strains isolated from coastal waters across different seasons and habitats.

Station	Genus	Summer	Autumn	Winter	Spring
St. A	*Thraustochytrium*	0	1	1	1
	*Oblongichytrium*	0	2	2	0
	*Sicyoidochytrium*	1	1	0	0
	*Schizochytrium*	0	0	0	0
	*Botryochytrium*	0	0	0	0
	Unidentified Thraustochytrids	2	0	0	0
	Total	3	4	3	1
St. B	*Thraustochytrium*	0	0	1	3
	*Oblongichytrium*	0	1	3	1
	*Sicyoidochytrium*	0	1	0	0
	*Schizochytrium*	0	0	0	0
	*Botryochytrium*	2	0	0	0
	Unidentified Thraustochytrids	0	1	0	0
	Total	2	3	4	4
St. C	*Thraustochytrium*	6	3	7	3
	*Oblongichytrium*	1	5	1	2
	*Sicyoidochytrium*	1	0	0	0
	*Schizochytrium*	2	0	0	0
	*Botryochytrium*	1	0	0	0
	Unidentified Thraustochytrids	2	0	0	0
	Total	13	8	8	5

**Table 2:** Fatty acid composition of thraustochytrids isolated from coastal waters across different seasons and habitats.

Fatty Acid	Relative Fatty Acid Content (%) ^#^
C12:0	1.59 ± 1.78
C14:0	2.12 ± 1.76
C15:0	5.81 ± 2.88
C16:0	18.42 ± 5.57
C17:0	1.31 ± 1.84
C18:0	7.68 ± 5.03
C18:1	4.75 ± 3.84
C20:0	3.48 ± 2.66
C20:4, w − 6	1.45 ± 1.30
C20:5, w − 3	3.89 ± 2.79
C22:5, w − 3	3.63 ± 4.79
C22:6, w − 3	25.14 ± 9.77

^#^ Mean ± SD are reported for *n* (number of strains) = 58.

## Data Availability

The 18S rRNA gene-sequencing data are available in NCBI’s GenBank database under the accession numbers OM418567–OM418624.

## Supplementary Materials

The following supporting information can be downloaded at: https://www.mdpi.com/article/10.3390/md20040229/s1, Figure S1: (a,b) Colonies of thraustochytrid isolates YC738 and YC719 on Petri dishes and (c,d) cells of YC738 and YC719 isolates as observed under a microscope; Table S1: Environmental parameters of the sampling stations.
