# Supplementary material for: Culturable Diversity of Thraustochytrids from Coastal Waters of Qingdao and Their Fatty Acids
Source: Mar Drugs. 2022 Mar 28;20(4):229. doi: 10.3390/md20040229 (PMC9029807; doi:10.3390/md20040229)
Supplement: Supplementary file 1 [file marinedrugs-20-00229-s001.zip › marinedrugs-1625909-supplementary.pdf]

Supplementary Information

Culturable Diversity of Thraustochytrids from Coastal Waters of Qingdao and their Fatty Acids

Mohan Bai [1,2], Biswarup Sen [2], Shuai Wen [2], Huike Ye [2], Yaodong He [2], Xiaobo Zhang[1,*], Guangyi Wang [2,3,4,*]

[1]College of Life Sciences, Zhejiang University, Hangzhou 310058, China

[2]Center for Marine Environmental Ecology, School of Environmental Science and Engineering, Tianjin University, Tianjin 300072, China

[3]Key Laboratory of Systems Bioengineering (Ministry of Education), Tianjin University, Tianjin 300072, China

[4]Center for Biosafety Research and Strategy, Tianjin University, Tianjin 300072, China

Keywords: Thraustochytrids, Diversity, Biomass, Fatty acids, Seasons, Habitats

*** Corresponding author:**

E-mail: zxb0812@zju.edu.cn; gywang@tju.edu.cn

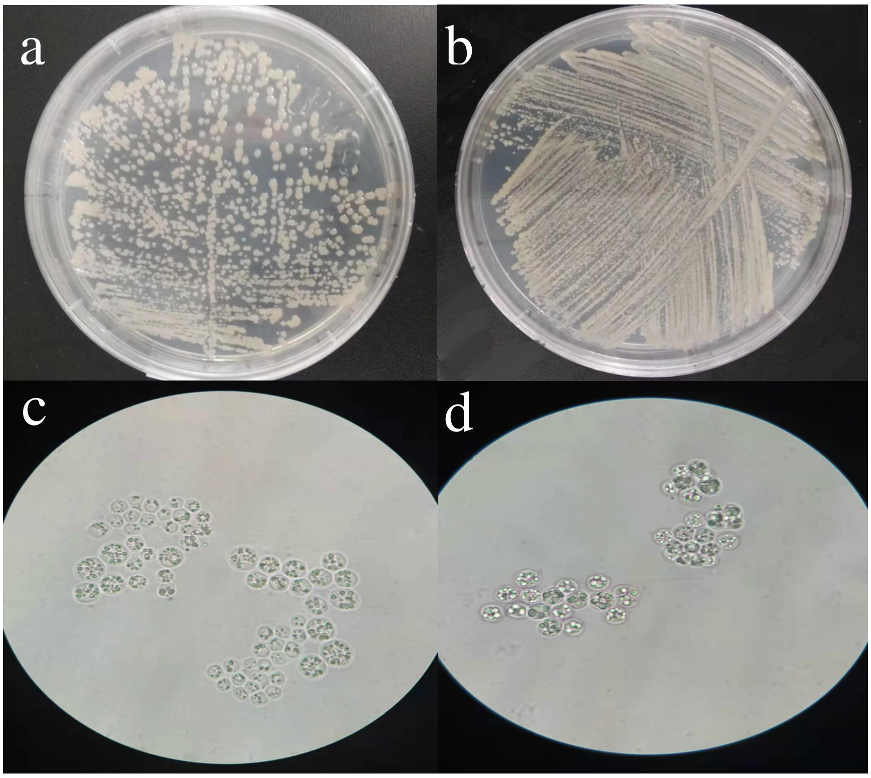

Figure S1. (a–b) Colonies of thraustochytrid isolates YC738 and YC719 on Petri dishes and (c–d) cells of YC738 and YC719 isolates as observed under a microscope.

Table S1. Environmental parameters of the sampling stations.

Station	Season	Temperature (℃)	Salinity (ppt)	pH	TP（μg/L）	TN（μg/L）
St. A	Spring	15.4±0.08	30.14±0.02	8.12±0	41.98±0	67.13±0.67
St. B		15.7±0.22	30.64±0.04	8.08±0.04	40.95±0	51.78±0.12
St. C		15.53±0.19	30.7±0.01	8.12±0	40.63±0.05	84.56±0.56
St. A	Summer	28.3±0.24	29.54±0.48	8.06±0.05	41.82±0.08	72.87±0.63
St. B		27.67±0.47	29.73±0.08	7.97±0.12	46.46±0	62.62±0.47
St. C		27.87±0.12	29.67±0.15	7.72±0.16	50.73±0	97.06±0.41
St. A	Autumn	18.1±0.08	21.47±0.29	8.07±0.04	45.75±3.48	93.45±0.65
St. B		18.63±0.05	29.94±0.04	7.99±0.07	41.05±0	66.01±1.02
St. C		18.33±0.12	29.54±0.02	8.02±0.02	42.29±0	147.26±0.72
St. A	Winter	5.57±0.34	27.63±0.21	8.11±0.02	39.95±0.02	113.85±0.41
St. B		5.2±0.08	27.6±0.16	8.06±0.04	40.05±0	96.27±0.51
St. C		5.17±0.05	29.94±0.04	8.11±0.04	41.22±0.02	128.52±0.51

Table S2. Seasonal changes in the lipid content (DCW%) of two dominant genera of thraustochytrids.

Season	*Oblongichytrium*	*Thraustochytrium*
Summer	7.29 (N=1)	9.21 ± 11.50 (N=6)
Autumn	36.84 ± 16.15 (N=8)	30.86 ± 15.98 (N=4)
Winter	18.61 ± 15.60 (N=6)	18.38 ± 8.98 (N=9)
Spring	11.25 ± 3.12 (N=3)	8.62 ± 7.13 (N=7)

Mean ± SD are reported.

Table S3. Biomass of different genera of culturable thraustochytrids.

Genus	Biomass (g/L)
Thraustochytrium	1.69 ± 1.16 (N=26)
Sicyoidochytrium	3.24 ± 1.05 (N=4)
Schizochytrium	2.95 ± 0.07 (N=2)
Oblongichytrium	2.41 ± 1.36 (N=18)
Botryochytrium	1.23 ± 1.20 (N=3)
Unclassified Thraustochytrids	1.28 ± 1.02 (N=5)

Mean ± SD are reported.
